# Transfer learning: a friendly introduction

**DOI:** 10.1186/s40537-022-00652-w

**Published:** 2022-10-22

**Authors:** Asmaul Hosna, Ethel Merry, Jigmey Gyalmo, Zulfikar Alom, Zeyar Aung, Mohammad Abdul Azim

**Affiliations:** 1 Department of Computer Science, Asian University for Women, 20/A M. M. Ali Road, Chittogram, Bangladesh; 2 Department of Electrical Engineering and Computer Science, Khalifa University, Abu Dhabi, United Arab Emirates

**Keywords:** Machine learning, Transfer learning, Multi-task learning, Sample selection, Domain adaptation, Zero shot translation, Image classification, Sentiment classification

## Abstract

Infinite numbers of real-world applications use Machine Learning (ML) techniques to develop potentially the best data available for the users. Transfer learning (TL), one of the categories under ML, has received much attention from the research communities in the past few years. Traditional ML algorithms perform under the assumption that a model uses limited data distribution to train and test samples. These conventional methods predict target tasks undemanding and are applied to small data distribution. However, this issue conceivably is resolved using TL. TL is acknowledged for its connectivity among the additional testing and training samples resulting in faster output with efficient results. This paper contributes to the domain and scope of TL, citing situational use based on their periods and a few of its applications. The paper provides an in-depth focus on the techniques; Inductive TL, Transductive TL, Unsupervised TL, which consists of sample selection, and domain adaptation, followed by contributions and future directions.

## Introduction

People can hardly afford the luxury of investing resources in data gathering in today’s world since they are rare, inaccessible, often expensive, and difficult to compile. As a result, most people found a better means of data collection: one of the ways is to transfer knowledge between the tasks [1]. This philosophy has inspired Transfer Learning(TL): to improve data gathering and learn in machine learning (ML) using the data compiled before it has been introduced. Most of the algorithms of ML are to predict future outcomes, which are traditionally in the interest of addressing tasks in isolation [2]. Whereas TL does the otherwise, it bridges the data from the source and targets the task to find a solution, perhaps a better one.

**Fig. 1:**
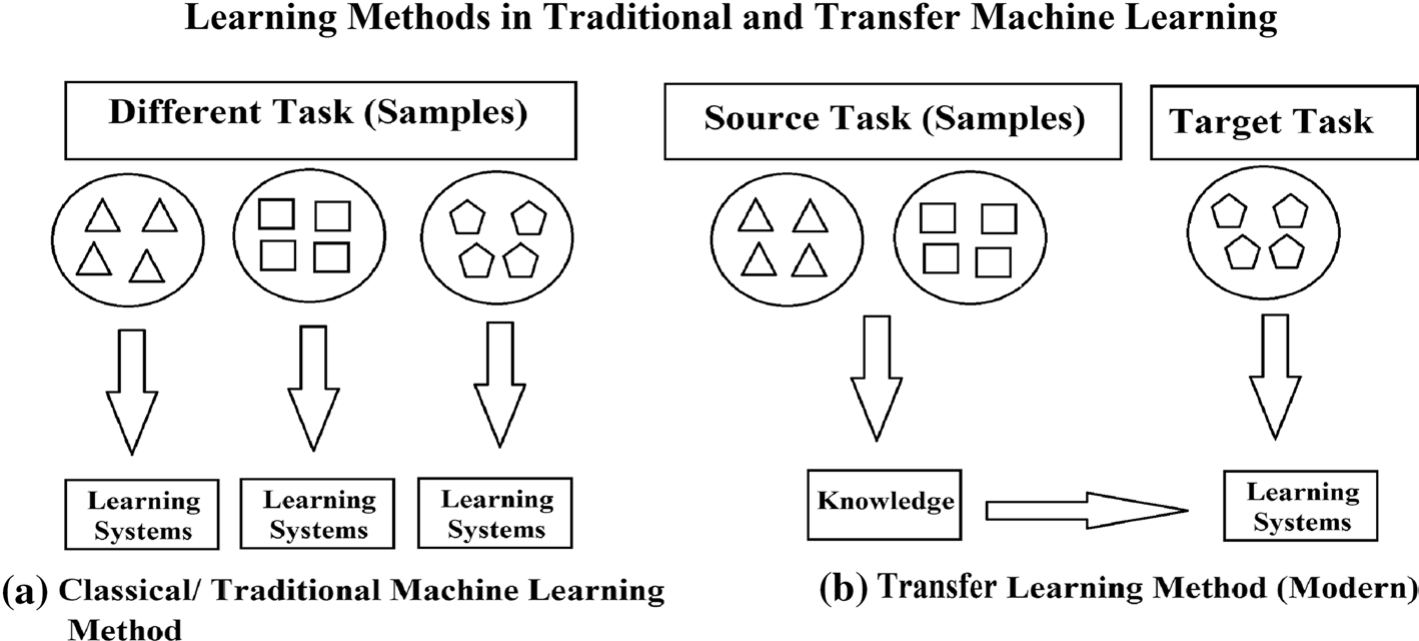
Traditional/Classical ML vs. TL [3]

TL aims to improve understanding of the current task by relating it to other tasks performed at different periods but through a related source domain. Figure 1 explains the improvement brought by using the TL strategy in ML. It enhances learning by creating a relation between previous tasks and the target task, providing logical, faster, and better solutions. TL attempts to provide an efficient manner of learning and communication between the source task and the target task, making learning debatable [3].In addition, TL is most applicable when there is a limited supply of target training data. The strategic use of TL is that not only among the performed(ing) task itself but somewhat beyond and across other tasks [4]. However, the relationship between source and target task is sometimes not compatible. If the user transfers the testing and training samples, it decreases the target task’s performance; such a situation is a negative transfer and vice versa.

This paper introduces the traditional approach to TL, improvements in the modern approach, techniques, applications of TL, data gathering, challenges, and the future scope of TL. Although TL is used in numerous areas with its varieties, this paper focuses on a few in-depth areas to provide brief insights and appreciation. The remainder of this paper is organized as follows. "Related work" section provides background information about the TL, definitions, and notations. "Techniques of TL" section describes the three settings of TL strategies: Inductive learning—case studies on multi-task learning and self-learning, Transductive TL, Unsupervised TL—sample selection, its applications, and domain adaptation in TL; "Domain adaptation" section describes numerous TL applications in different domains. "Contributions of TL" section addresses some of the contributions made by TL in medical and related fields. "Future directions of TL" section provides the future directions of TL techniques and conclusions, respectively.

## Related work

To date, the disciplines of traditional ML and data mining have been extensively applied in many areas, such as retrieving patterns from existing records obtained from labeled or unlabeled data sets, for instance, training data, to predict future occurrences [5]. Traditional ML uses training and testing data methods with similar data distribution and input featured. Following the difference generated in the distribution data between the training and the testing set, the outcome or the prediction can either be deteriorated or improved [6]. In some cases, acquiring training data that fits the testing data’s input feature set as well as the anticipated outcome of distribution data features can be quite challenging and very costly [2]. As a result, a top-level learner is required for any target domain, which has previously learned and improved from a related field. This innovation drives the backbone of how TL is being adopted today.

TL focuses on wide domains, tasks, and patterns in both training and testing datasets [3]. Multiple instances of TL can be seen in the real world, such as the ability to distinguish between objects like cars and bikes. Another real-life example can be two individuals learning how to ride a bike. Assuming that one person has no prior biking experience, while the other person has some practice of riding a bicycle. In that situation, the person with the bicycle background will be able to learn to ride a bike comparably faster than the other person since his prior understanding of riding a bicycle will aid him in learning the task of riding a bike effectively. Likewise, TL operates on the premise of storing information from a previously learned task and applying it to a new one. The idea of TL is driven by the fact that humans can effectively relate previously acquired skills in solving contemporary challenges quicker and more accurately [3].

Since 1995, a variety of terms have been used to describe TL studies, some of which include “learning to learn,” “transfer of knowledge,” “multi-task learning,” “inductive transfer,” “knowledge integration,” “knowledge-based inductive bias learning,” “supervised learning,” “meta-learning,” and “semi-supervised learning” [3, 7]. Among such, the multi-task learning model is seen to have a strong learning strategy that is similar to TL because both learning models strive to learn multiple tasks at the same time, despite the fact that they are different [8]. A detailed and insightful approach to multi-task learning is explored under the TL technique in the latter part of this study. Figure 1 depicts the differences between traditional ML and modern TL strategies in effective learning. As shown, classical ML only attempts to learn from the root/scratch, whereas TL aims to transfer information from the primary tasks to a new task with a top-quality training data set.

Additionally, as mentioned above, TL is required when the target training set is scarce. This can happen due to data problems like the rare, costly to acquire and evaluate, or even unavailability of data. However, TL strategies have become more appealing as large-scale data sources grow increasingly accessible. Utilizing available datasets that are somehow related but not the same makes this learning approach such a viable method [2]. Some ML applications where TL has proved to be successful and discussed in this paper are TL in a real-world simulation, sentiment classification, gaming, image classification, and zero-shot translation.

Overall the paper considers providing a generic appreciation to TL, a technique of ML to enhance the performance between the training dataset through the acknowledgment of the trained dataset. Unlike other articles, this paper brings in the background, ongoing and future scope of TL, emphasizing multi-task learning, sample selection, and domain adaptation.

### Definitions of TL

According to Matt, he defines TL, a category under ML is when the reuse of pre-existing models to solve current challenges. He also acknowledges that TL is a technique employed to train models together. The concepts of pre-existing training data are utilized to enhance the performance of the ongoing challenge, so the solution need not have to be developed from scratch [9]. Similarly, Daipanja also aligns with the above definition of TL. He further uses the comparison between the traditional ML approach where the data were isolated based on specific tasks, and each challenge was developed from scratch, with limited knowledge to acknowledge one another. Now, however, the TL, the acknowledgment of previous data; trained models for the current training models have been comparatively enhanced and emphasized [10]. An article by Yoshua et al. defines TL as the technique that trains current models with trained models of previous similar related tasks. The explanations are wide and varieties of explanations are provided; however, most of them align with each definition. Lastly, Jason writes, TL is an optimization tool that escalates the performance of modeling second task [11].

### Relationship between ML and TL

The relationship between TL and ML can be understood when TL improves developing models through the pre-trained models and improves their efficiency. A few of the benefits of TL includes as follows: starting every task or current challenge by building new model to train and test; scratch, Improves the efficiency of ML techniques and models progression Relation between the dataset could be understood from different points of view rather than in isolated terms. Models could be trained based on the required simulations rather than the natural world environments. In times when the resources are limited and the observations of the models are required, TL is one of the tools that help in learning and generating more accurate results so the assigned target domain functions [9].

## Techniques of TL

This section covers the techniques used in TL’s core approaches to three major research questions: What, How and When to transfer.

- The question of “what to transfer” entails determining which aspects of information or knowledge will be shared or transferred between domains or tasks. Sometimes, some information may be specific to certain domains, while others are exchanged across common domains, which may increase performance in the target domain.
- Following the discovery of which information may be transmitted, learning algorithms to transmit the information must be devised, which now correlates to the questions of “How to transfer.”
- “When to transfer” question when it is appropriate to transfer the available information. In some cases, when the source and target domains are unrelated, the transfer may fail forcefully. Alternatively, under a worst-case scenario, it may even harm the learning performance of the target domain, a circumstance termed as “negative transfer.” For this reason, avoiding negative transfer is a critical unanswered question till today.

Based on distinct conditions between the source domain, target domain, and the tasks, there are three sub-settings of TL strategies, categorized as: inductive TL, transductive TL and unsupervised TL (see Table 1).

**Table 1:** TL strategies and its settings

Learning strat-egytarget tasks	Related areas tasks	Clustering dimensionality reduction	Target domains	Source domain labels	Target domain labels	Source
Inductive TL	Multitask learning	The same	Available	Available	Different but related	Regression classification
	Self-taught learning	The same	Unavailable	Available	Different but related	Regression classification
Unsupervised TL		Different but related	Unavailable	Unavailable	Different but related	Clustering dimensionality reduction
Transductive TL	Domain adaptation, sample selection bias, Covariate shift	Different but related	Available	Unavailable	The same	Regression classification

### Inductive TL

In this case, the target task differs from the source task, despite the source and target domains is similar. With traditional learning, the focus is usually on the target domain or tasks; however, in multi-learning or multi-tasks learning (a subset of Inductive TL), the goal is to excel at every task available [3]. Inductive TL is further classified into two cases based on its labeled or unlabeled data source:

#### Case 1—Multi-task learning

Here, the source and the target domain are the same, and a ton of labeled information in the domain source is accessible. For this situation, the ’inductive TL setting’ is similar to ‘multi-task learning’ since the source and target are the same. Notwithstanding, the inductive learning setting only targets high performance in the objective task by transferring information from the primary source. Multi-learning attempts to gain proficiency with the aim and source the job simultaneously.

#### Case 2—Self-taught learning

Here, the source and target domain are different but somehow related. No labeled information in the source area is accessible. For this situation, the inductive TL is similar to a self-taught learning setting, which implies that the spaces between the source and target areas materialize to be unique and somehow related to inductive TL (first reported by Raina et al [12]). Note that here the labeled information in the source area is inaccessible [3]

### Transductive TL

Here, both the tasks (source and target) are the identical in this case. However, the domains are distinct. As shown in Table 1, no labeled data is available in this target domain, although amany labeled data areavailable in the source domain.

### Unsupervised TL

In this TL scenario, the target and the source task are different but somehow related, similar to the inductive TL. Unsupervised TL, on the other hand, focuses more on completing unsupervised tasks, such as clustering and dimension reduction [13, 14]. In this situation, both the domains, i.e., source and target, have no labeled data.

## Sample selection

The sample selection in TL is one of the most critical areas of the building model workflow. It is where selecting variables, and source tasks take place concerning the target task’s requirements. There are primarily two factors to begin the sample selection. Considering these requirements, Firstly, the common sense, the user of sample selection should have an intuition that there is a relation between metrics of source variables that matches or is similar to the target task. The metrics that add value and quantify the target task are then chosen to begin with problem-solving tasks.

Second, to take caution, although there might be many promising source variables and domains available in the source task, the user should be aware of what metrics target task values. While selecting the most relatable and efficient data for the target task, an important issue is not adding too many parameters from the source task that will eventually cause overfitting. However, when overfitting occurs and sample selection negatively affects the target task, a penalty over data is introduced. Penalty criteria narrow the parameters to incorporate only the most helpful information target required from the source task [3].

### Sample selection bias

**Fig. 2:**
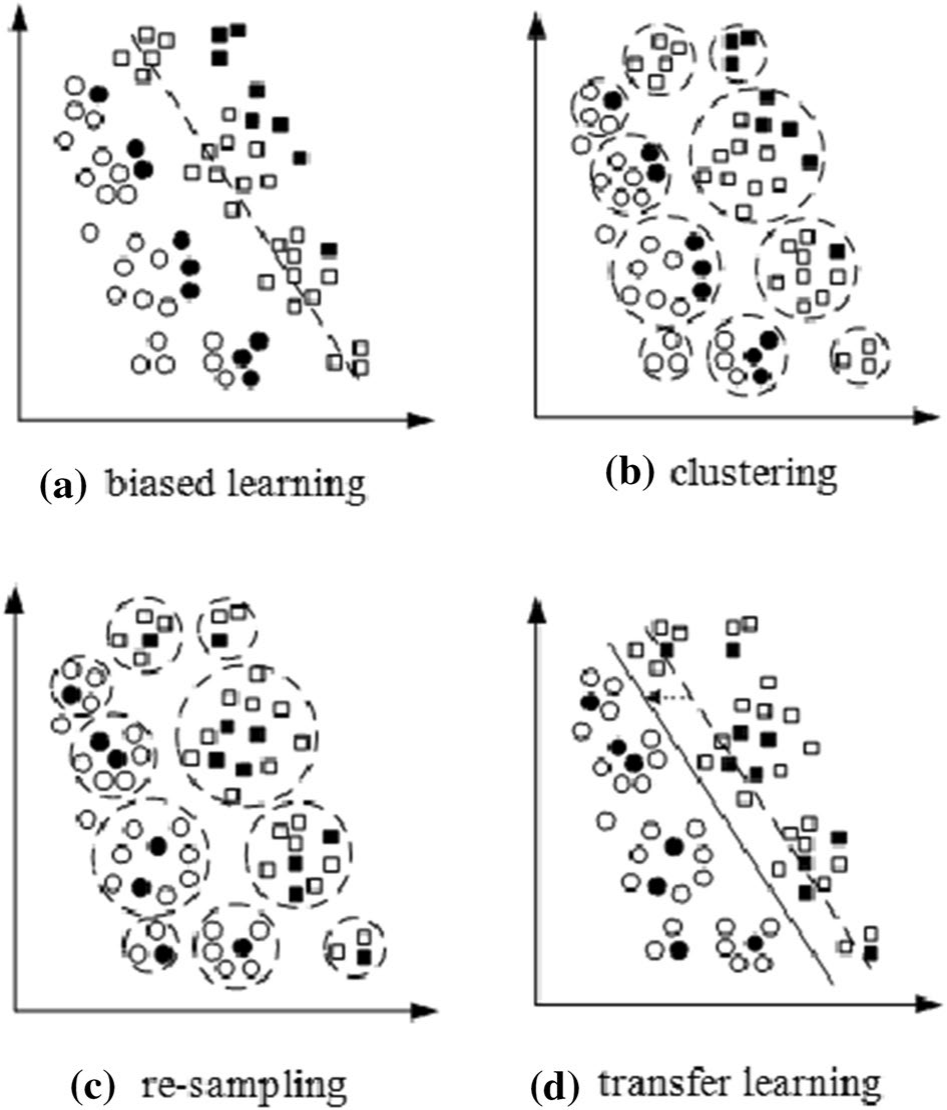
Changes brought by sample selection in TL

Sample Selection Bias has been acknowledged as one of the most complicated issues in practical application. The future, and the current training data *d* differ constraints and distribution [15, 16] . Thus, causing the side effects of sample selection [17]. In some cases, small sampled groups duplicate or create a pattern for bigger groups of the sample, eventually leading the datasets to suffer from sample selection bias. Nevertheless, some measures have been applied to reduce sample selection bias, especially between the target and source task. Figure 2 demonstrates the changes bought by sample selection in TL.

It can be expressed that many ML algorithms use almost the same training data to test data which will soon be used to make predictions on the training data. However, it fails to recognize that the practical applications use data that are sometimes different from one another, creating variations between the testing and training data. Conventionally, following the distribution of *Q*(*x*, *y*), the datasets are trained and similarly, the distribution $P(x, y) = Q(x, y)$ is employed on a dataset for testing. In the last few years, strategies to improve the sample selection area have been constantly enforced and several articles have been published focusing on this matter. Note that a compact overview of the TL strategies and their settings are given in Table 1.

Regardless of interventions in this area, sample selection has been susceptible to bias, including the choices that inappropriately select the control groups in the case–control studies, bias in loss-to-follow-up cases, and others. Like the other TL features, sample selection has received massive attention from the communities such as ML, statistics, economics, bioinformatics, epidemiology, medicine, and many others. Sample selections use source data to build to predict prediction and alter the source data. Such action crosses data limits to the broader range and beyond a single data distribution giving the user higher scope to build efficient models. It is one of the TL areas that has received much attention from ML and research groups in the past few years.

### Brief analysis of sample selection; Kernel mean matching algorithm

**Fig. 3:**
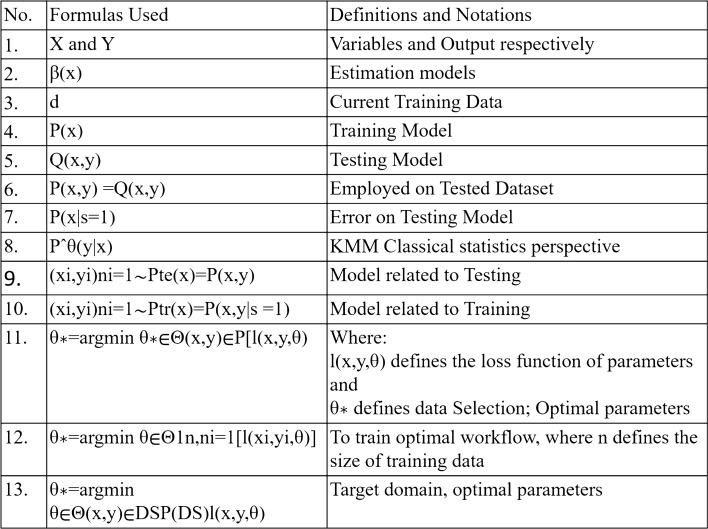
Symbols abbreviations [16]

Estimation models $\beta (x)$ such as kernel density estimation, a naive approach, are used to measure the training sample and minimize selection bias from the external data [18]. Regardless of using them in the label data distribution, some models have been inferior or less effective. Being less effective includes not estimating data with high density or data with heavy information. Also, if the model makes a small estimation error, in that case, especially when the testing $P(x|s = 1)$ and training *P*(*x*) models have small values, it disturbs the whole performance of the data causing relatively worse performance to the target task compared to source task. This incident was noticed in several cases of performance evaluation. Estimating $\beta (x)$ directly was considered more logical than evaluating these models while working with the target task having huge data density and less training and testing samples (Fig. 3).

Gradual improvements are made in the estimation models $\beta (x)$ present in the covariate shift; an algorithm is suggested known as kernel mean matching (KMM) and unconstrained least-square importance fitting (uLSIF) [1]. However, only the KMM model will be elaborated in this paper although, we acknowledge the unconstrained least-square importance fitting (uLSIF) and its uses.

Both of them are better measures and versions of the kernel density estimation. The KMM model considers the classical statistics perspective, denoted by $P^{\theta (y|x)}$. It is a parametric model that organizes the label data distribution, mainly for the logistic regression models, and applies to other models. It estimates the prediction loss from the source data to the target data and reduces its overfitting. A form of penalty criteria is to narrow the parameters to incorporate only the most helpful information target required from the source task.

The KMM also contradicts the primary assumption of ML, which stated that the testing and training of data comes from one data distribution. The model relates the testing ${(x_i , y_i )}n_i=1 \sim Pte(x) = P(x, y)$ and training ${(x_i , y_i )}n_i=1 \sim Ptr(x) = P(x, y|s = 1)$ samples from multiple sources and ultimately predicts how *X* (variables) equals to the *Y* (output).

Data sampling falls under transductive TL, which helps to learn an optimal model workflow for the target domain and task by minimizing any expected risk. Concepts of empirical risk minimization (ERM) are some of the measures that help stimulate data and its risk towards the target problem. Thus, optimal parameters ($\theta ^{*}$) such as; $\theta ^{*} = arg min \ \theta ^{*}\in \Theta (x,y) \in P [l(x, y, \theta )]$ , where $l(x, y, \theta )$ defines the loss function of parameters. Similarly, since estimating the probability distribution of data is difficult, the ERM concept is then utilized. To train the optimal workflow; $\theta ^{*} = arg min \ \theta \in \Theta 1 n, n_{i=1} [l(x_i, y_i, \theta )]$, where *n* defines the size of training data [19].

The above models for workflow are used in source data selection, but the target domain does not remain ideal. Optimal parameters such as; $\theta ^{*} = arg min \ \theta \in \Theta (x,y) \in DT P(DT )l(x, y, \theta )$. However, the training dataset should be obtained from the source domain to structure the target domain. In the case of $P(DS) = P(DT)$, we can solve optimization using the target domain. $\theta ^{*} = arg min \ \theta \in \Theta (x,y) \in DS P(DS)l(x, y, \theta )$.

In contrast, if *P*(*DS*) is not equal *P*(*DT*), modification of the above optimization problem is considered where a model learn high generalization ability for the target domain, as follows: Several methods are available to predict the values of *P* ($xS_i$) *P* ($xT_i$). According to the Zandrozny [20] , the author requested to estimate the numerical values of $P(xS_i)$ and $P(xT_i)$ without depending on the other classification problems. Similarly, an article by Fan et al. [21] elaborated this concept and idea of solving selection problems by using multiple classifiers to predict the probability ratio.

Lastly, the covariate shift or sample selection bias offers a considerable advantage and exponentially increases data quality to process the target data.

### Applications of sample selection

In TL, sample selection has been used in several areas and models, making them different. It has been used to study drugs at the medical clinics, choose patients from the general population of the given demographic, and many others. The selected data thus differs from the general population based on gender, race, and patients. It has also been used in system detection software built many years ago to improve its predictive method and capabilities.

After using sample selection, the old system lacked organizing due to modern times’ new attacks or spam patterns that improved these challenges. The surveys based on particular religions that were undertaken did not relate to others due to their differences also used sample selection to link and bridge and apply to one another types of research which otherwise. Overall toward the survey’s religion, sample selection has been used to bring about relations among them with the other categories of the beliefs. All of the given examples do not follow the primary assumption of ML, which states that training and testing data have to be from one data distribution. The data are of more than one data distribution, where testing and training occur in different domains. Such selection makes the sample selection users thoughtfully choose the standard features of previous source data and the current target data.

Considering the above applications and the sample selection usage, it defines how many datasets in real-world applications are potentially biased. Further research has been done based on the sample selection bias. The proposed approaches do not assume the exact type of biases or formal models to quantify the distribution of the bias to rectify [20]. Reducing the sample selection bias is an open problem.

## Domain adaptation

Domain adaptation is a type of TL in which the task remains the same but the source and destination have different domains or distributions. Consider a model that has been trained on *x*-rays of many patients to determine whether or not a patient is infected with covid-19 [22]. However, the best-generalized systems depend on appropriate datasets [3]. If the data is biased, the system can not generalize accurate outputs, and such a problem set is known as domain adaptation. Domain adaptation helps apply an algorithm to train one or more source domains to improve the target domain. The Domain adaptation process tries to alter the source domain to bring closer the distribution of the source domain to the target domain [23].

### Mathematical explanation of the domain adaptation

Lets denote the domain as *D* which contains two components; a feature space *X* and a marginal probability distribution *P*(*x*). Feature space *X* can be $X_1, X_2 ..... X_n .... \infty$.

So, supervised learning tasks on a specific domain will be, $D=\{X,P(x)\}$. Further task will be consistent with *Y* lebel space and object predictive function *f*(); it will be denoted as $T=\{Y, p(y/x)\}$. The predictive object function *f*() may train data which contains the pairs of ($x_i$, $y_i$); where *Y* can be $y_1$,$y_2$, $\infty$ and function *f* is used to predict the level of *x*. However, domain adaptation has two domains and two tasks. Given source domain [4], $D_S=\{XS,p(xs)\}$, where $XS={xs_1,....xS_n}$ task on the source domain, $T_S =\{YS,p(ys/xs)\}$, where $YS=\{YS_1, ... YS_N\}$ target domain $D_T= \{XT,P(XT)\}$, where $XT=\{XT_1\ldots .XT_N)\}$ task on target domain $T_t= \{T_t, P(YS/XT)\}$, where $YT=\{YT_1 ... YT_n\}$ [24] Those components are defined as improving the learning of the target predictive function $f_T()$. The target domain uses the information of the source domain and task on the source domain. $f_{ST}()$ is initiate as a predictive model to train the source domain $D_S$ so that that domain can adopt the target domain $D_T$ [23].

## TL applications

### Real-world simulations

Large production companies face enormous challenges to be more flexible for any production fluctuation while providing higher product quality with significantly lower costs in manufacturing and processing in a dynamic workforce [25]. The central objective of this process’s underlying setup is to distinguish optimal parameters that fulfill high product quality and efficiency.

One approach to defeating these difficulties is exploiting the techniques of artificial intelligence (AI), mainly supervised learning models. Supervised learning trains the models by using appropriately categorized or labeled information. Each ML (ML) or AI application that depends on gathering information or preparing a real-world model is costly, tedious, or even hazardous for our use or the environment. Therefore, robots are being trained to utilize simulations results in advancement and technology and limit development costs. Consequently, with these advancements, the systems become more practical and ideal. Furthermore, one can train, test, recreate, and program the robots to train themselves so that the real-world robots can transfer and use each information learned in the process. These kinds of transfers are done using progressive networks, an ideal platforms for real-world robot simulations. Contrarily, sometimes not all the simulation highlights are effectively repeated when applied in the actual word because of their complex interactions.

Considering the enhancement of the performance, the TL techniques have been also emphasized and used in the real world simulation dataset. The dataset and research articles based on real world simulation include; Policy transfer from simulations to the real world by transfer component analysis [26], Simulations, learning and real world capabilities [27], Real-world reinforcement learning via multifidelity simulators [28], Knowledge-aided Convolutional Neural Network for Small Organ Segmentation [29], Adaptive Fusion and Category-Level Dictionary Learning Model for Multi-View Human Action Recognition [30] and Stimulus-driven and concept-driven analysis for image caption generation [31] to name a few.

### Gaming

The rise in ML and other AI applications has made an enormous impact on gaming advancements so far. Today, one of the fine examples of this yield is AlphaGo, one of the first ML programs that defeated an expert human Go player, developed by Deepmind’s neural network. AlphaGo is an ace in this game. However, it is incompetent with other games and fails to win when entrusted to play different games. This failure happens because it is only programmed, designed, and fitted to play ’Go,’ which drives the ultimate drawback of utilizing artificial neural networks (ANN) in gaming. It can neither be as fast nor ace all games like a human brain. Therefore, in order to play and win other games, AlphaGo needs to thoroughly forget the algorithms of ’Go’ and learn to adapt to an entirely new program [32].

Consequently, with the help of TL, new games can now be played by re-applying the strategies learned in a previous game, as the definition of TL states. For instance, the applications of TL in gaming can be seen in the game MadRTS [33], which is a real-time strategy game that includes ongoing competing players. In this game, the application uses CARL (case-based reinforcement learning (RL)) [34], which is a multi-layered plan that joins case-based reasoning (CBR) and Reinforcement Learning (RL), that permits us to secure as well as separate the keys and strategies of our tasks and use the particular idea of TL in it [33].

### Image classification

Multiple models on image classification have been developed to facilitate the resolving of the most pressing issue of identification and recognition accuracy [35]. Image classification is a significant subject in computer vision, with many applications. Object identification for robotic handling, human or object tracking for autonomous cars, and so on are a few examples of the applications of image classification [35]. Today, convolutional neural networks (CNN) show reliable outcomes on image or object detection and recognition that are helpful in real-world applications [36]. The architecture of CNN models works on training and predictions on a high level of abstraction. One of the best tendencies of neural networks is the ability to perceive things inside an image as they are prepared on labeled pictures of massive datasets, which is very challenging in time management. Several Computer Vision and ML issues have demonstrated that the CNN framework performs effectively on solving accuracy.

**Fig. 4:**
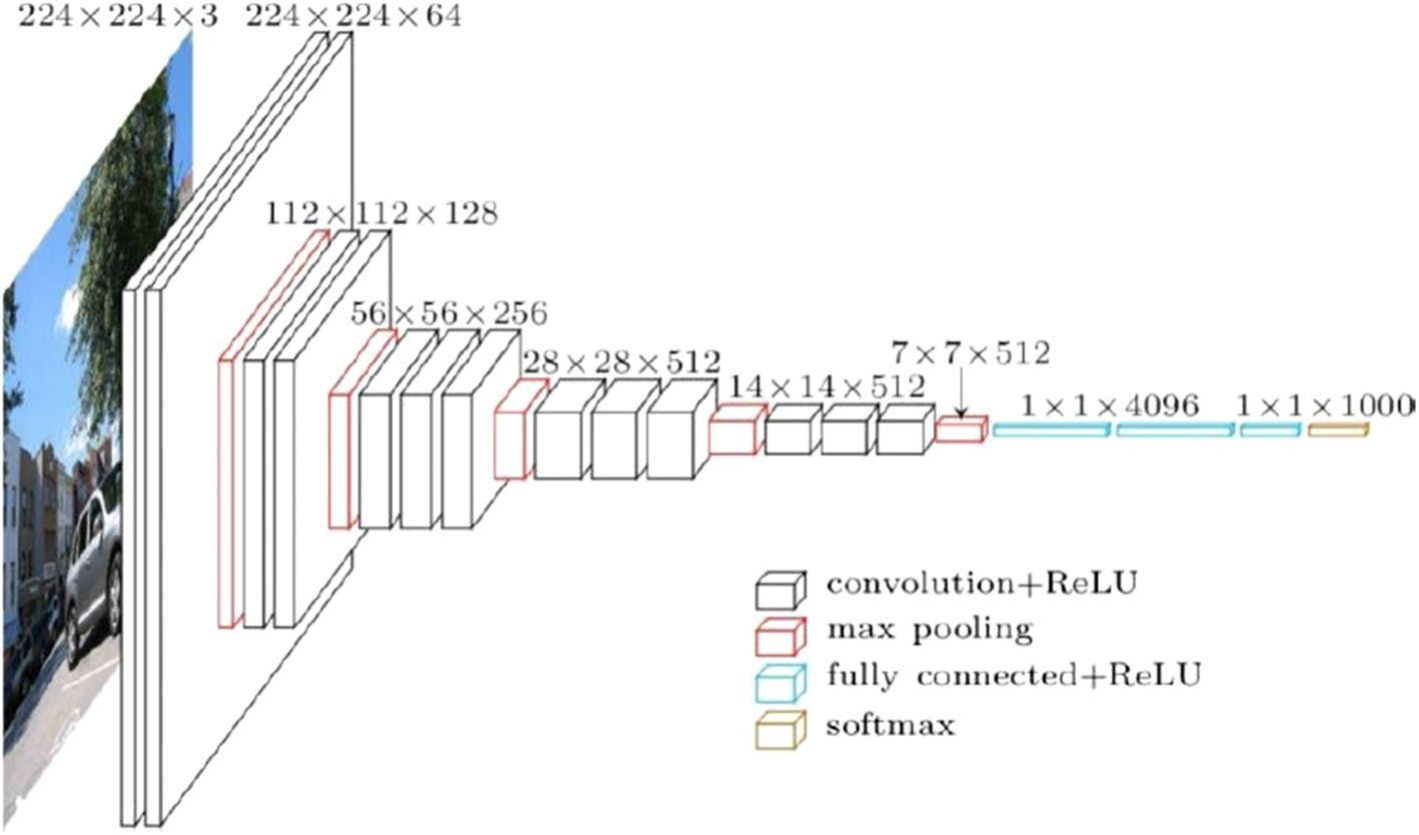
The architecture of VGG-16 ConvNet [39]

Convolutional Neural Networks (CNN) have influenced and dominated the ML vision field. In recent years CCN comprised three layers, namely, “an input layer, an output layer, and several hidden layers that includes deep networks, pooling layers, fully linked layers, and normalization layers (ReLU)” (main). For example, in a VGG-16 [37] ConvNet [38], illustrated in Fig. 4, that consist of different layers containing a unique collection of picture combination attributes. The figure must be prepared to perceive images inside a dataset. In doing so, it is firstly pre-trained by utilizing ImageNet. It is layer-wise ready, beginning from the SoftMax layer and preparing it simultaneously, followed by the thick layers. However, these models rapidly strain battery power, limiting smaller gadgets, storage devices, and inexpensive phones [36, 39]. To reduce such burdens, TL helps prepare the models through pre-training using ImageNet, consisting of many pictures from various sources and saving time. Another example can be, if a facial image is set as the input into a CNN structure, the system will start to learn basic properties in its training stage, such as lines and edges of face, bright and dark areas, contours, and so on [35].

### Zero shot translation

One of the popular procedures for machine translation is the neural machine translation (NMT) [40] which a colossal artificial neural organization achieves. It has displayed promising results and has indicated tremendous potential in unraveling machine interpretation and translations. The best way to exercise machine translation into a language can be done with a touch of planning data using zero-shot translation [41]. In this note, Zero-shot learning is considered as one of the most promising learning strategies, where the input sources and the classes we intend to portray are disjoint. Accordingly, zero-shot learning is connected to using supervised learning (similar to applications in gaming) to access its accuracy and the training data. One famous example is Google’s Neural Machine Translational model (GNMT), which considers powerful cross-lingual interpretations. For instance, to translate two different languages, Korean and Spanish, we need to have a pivot language (intermediary language) representing the two dialects. Firstly, Korean must be initially translated into English and later to Spanish. Here, English is an intermediary between Korean and Spanish, known as the pivot language.

Therefore, to avoid all the turns and bends from one language to the other, zero-shot can utilize all the available data to understand the translational data applied and to decipher it into a new translational language [42].

### Sentiment classification

Understanding hidden or visible feelings conveyed online or in social media is essential to customers, and users [43]. Sentiment classification is acknowledged as perhaps the most significant area in Natural Language Processing (NLP) research. Social media has surpassed as the essential way of generating opinion data and because of domain diversity, applying sentiment classification on social media has a great deal of potential, but it also has many challenges [44]. One of the most common sub-areas in sentiment classification is interpreting an individual’s feelings conveyed via media content. Sentiment classification of social media data is unquestionably a project of big data. Earlier research based on sentiment classification analyses texts within a linguistic expression.

**Fig. 5:**
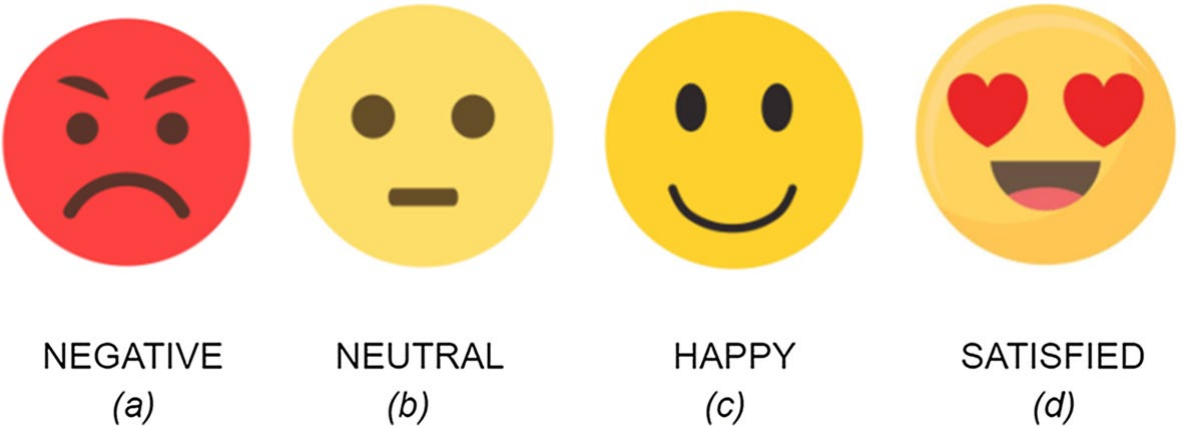
Polarities of sentiment analysis [44]

Sentiment classification is an additional helpful tool that allows a user or any business organization to identify and know their client’s choices and reviews by understanding their sentiment based on negative, positive, or neutral reviews (as Fig. 5), which may also be labeled as good/bad, satisfactory/not satisfactory. It is tough to build an entirely new compilation of texts to analyze sentiments since it is not easy to prepare models for identifying their feelings. Therefore, a solution to these problems can be solved using TL. For instance, if *x* is the input text and *y* is the feeling or thought, we need to predict a film review. The deep learning models are prepared on *x* input via sentiment analysis of the content corpus and identifying every statement’s polarity. When the model is prepared to understand feelings through the extremity of *x* information, its basic language model and learned knowledge is then transferred onto the model allotted a task to examine sentiments to *y*, i.e., film reviews. Additionally, different techniques such as embedding are also used in identifying various jobs related to sentiment analysis by transferring information from one source (*x*) and re-applying the same algorithms in the targeted area (*y*) to fulfill the predicted task [45].

Figure 5 shows the polarity of sentiment analysis Unhappy with the service Neither happy nor sad Happy with the service Very happy and totally in love with the service.

## Contributions of TL

### Contributions in medical sciences

Medical imaging and MRI play essential roles in routine clinical diagnosis and treatment. In MRI the variation shows the difference between standard and disease tissue. With ML and TL knowledge, medical scientists can easily detect the disease. However, there are vast training data that can be expensive to utilize. So, TL uses medical imaging and makes it Convolution neural networks(CNN) are a great success in analyzing medical images and making variations in medical imaging protocols. TL shows outstanding performance in white matter hyperintensity (WMH) segmentation (vascular origin, which can find on brain MRI or computed tomography), tumor segmentation (detecting the location of tumor cells), microbleed segmentation (detecting traumatic brain injury). Therefore, in many cases, network training on one MRI data acquired does not work efficiently in other protocols [46]. However, domain adaptation helps to ensure the usability of trained models in actual practices. This limit can be solved using the trained models with large annotated legacy datasets on new datasets with different domains, and trained models will require the clinical setting [47]. MRI shows the high variation among soft tissue and contrasts. For example, the image database, Imagenet contains more than fourteen million annotated images with more than twenty thousand categories. From them, finetune is based on the instances from the target domain [48].

### Contributions in bioinformatics

In the bioinformatics field, analyzing biological sequences plays an important role. To understand the organism’s function, biologists should analyze the gene sequence of the particular microorganisms. However, TL and domain adaptation show outstanding bioinformatics performance (i.e., gene expression analysis, sequence classification, and network reconstruction). Domain follows different models of organisms or different data-collecting sets to maintain the predicted sequences. If there is any error while predicting the series, prognosis systems alarms to replace the component, and the system changes its properties. It will alarm until the system changes or is replaced by a new setting and component [46]. However, the bioinformatics application has many different problems of distribution. For example, two organisms’ functions can be the same, but the substance can differ, let in marginal distribution differences. On the other hand, if two species belong to a common ancestor with long evolutionary history, in this case, conditional distribution difference will work significantly. The TL can be used to predict the mRNA splicing. In the mRNA splicing case, a source domain can be C.elegans organism, and the target domain is C.remanei and P.pacificus organism. Several TL approaches compare, i.e., FAM and the variant KMM can improve classification performance. Besides, gene expression analysis predicts the association between genotypes and phenotypes. However, in this case, it can have data sparsity problems(not observing enough datasets) as the nucleotides sequence is minimal data. To ensure outstanding performance, TL can be used to provide other information [48].

There have been numerous use of TL in bioinformatics; the dataset uses the algorithms of TL to showcase the contribution of TL in its section, respectively. Few of the samples of dataset includes; TL for BNER Bioinformatics 2018 [49], Exploiting TL for the reconstruction of the human gene regulatory network [50] , Parasitologist-level classification of apicomplexan parasites and host cell with deep cycle TL (DCTL) [51], AITL: Adversarial Inductive TL with input and output space adaptation for pharmacogenomics [52], Improved RNA secondary structure and tertiary base-pairing prediction using evolutionary profile [53], mutational coupling and two-dimensional TL where the Computational prediction of RNA secondary structure is performed with reference of TL, Optimized hybrid investigative based dimensionality reduction methods for malaria vector using KNN classifier [54], A survey of dimension reduction and classification methods for RNA-Seq data on malaria vector [55] , A hybrid heuristic dimensionality reduction methods for classifying malaria vector gene expression data [56] and others to follow. https://www.overleaf.com/project/61fd387cb8f6c7ccd64510aa

### Contributions in transportation

TL has transportation applications, i.e., understanding the traffic scene and target driver behavior. Here, images are taken from specific locations [57]. However, the outputs could be incorrect in variation because of different weather conditions. TL can give an outstanding solution by pictures taken in the exact location at different weather conditions [23]. For this, firstly, the system trains the network to specify the feature of pictures. Secondly, a new feature is built by the feature transformation strategy. Then, the dimension reduction algorithm generates a low-dimension feature. However, in the last stage, among the tested image, recovered best-suiting image and the Markov model transfers, the cross-domain sets with a best-matching image to test [58]. TL can be applied in target driver behavior with sufficient personalized data of each target driver’s behavior. TL can demonstrate the result even when target domains are limited, and data are very small or very large [48].

### Contributions in the recommendation system

In this contemporary world, the most heated topic for many industries is to build up an automatic question answering system that more likely works as a recommendation system. Recommend er systems are widely used in the e-commerce field. It helps people to answer all questions that are related to the merchandise [59]. E-commerce has been an essential part of everyday life for people, and they are familiar with this field. Based on different products, or services, it has different websites where people can shop. E-commerce can be divided into vertical e-commerce and integrated e-commerce websites. From vertical e-commerce websites, people can shop for the same sorts of products. In contrast, integrated e-commerce websites sell multiple products, including food, clothing, research service, etc. The e-commerce recommender system used three techniques to recommend products, i.e., collaborating filtering, content-based filtering, and hybrid recommendation. According to information retrieval, content-based filtering first analyses consequences obtains a set of features, and then builds product feature vectors. Then it calculates the similarity between users and products then recommends. In ML, clustering is used for content-based filtering. Secondly, collaborative filtering recommendation follows two techniques: memory-based filtering and model-based filtering. Memory-based algorithms work on users’ ratings and preferences for a particular product. It predicts the target product of a user. Nevertheless, initially the memory-based and model-based algorithms study according to the ratings of records as well concerning the target user’s rating. Hybrid recommendation works the same as a content-based recommendation, but it can better perform than collaborative filtering. Though recommendation application did a lot in e-commerce, it has been facing many data sparsity problems, leading to poor recommendations [60]. However, TL comes out with the best recommendation system that combines collaborative filtering proposals to alleviate sparsity problems. This method improves the accuracy of advice by transferring the knowledge learned from dense data sets to sparse ones. TL makes the new framework for e-commerce recommender systems in which knowledge is known from the source domain and source task target domains and target tasks. With the help of TL, people can use knowledge and can solve their problems faster [61].

## Future directions of TL

A great future is awaiting further advancements in TL research. We find many modern visual learning algorithms on data, those of desired object categories. For instance, the Object-oriented paradigm algorithmically detects, recognizes, and describes the unseen images [48]. We need new data collections containing the precious label to execute those modern visual learning algorithms. However, many pre-existing large datasets, such as Imagenet, have about 150M images, a massive pool with more information.

TL aims to use the previous knowledge and related source task and emphasize extra source data to boost a poorly targeted set [2]. Besides, TL problems can be solved by dynamic settings for online learning and self-leveling data. Therefore, most often, pre-existing resources are ignored because of no overlapping. This pre-existing can be used for classification and localization; no past knowledge and datasets are useless. Therefore, we can contribute to taking some steps to ensure the best use of pre-existing data. We can revisit the past knowledge and generalize, which may make research potential and relevance for practical purposes and application [46]. Secondly, we may improve the previous TL methods when the least annotated samples of the new target domain are available. Zero-shot classification is the advanced step because it obtains classifiers for novel categories and arbitrary basis though less data is available. Besides, zero-shot is reliable for the textual embedding of image datasets, and it is faster, more accurate, and more economically active. Finally, we may combine the zero-shot and active learning in support vector machines with optimal query conditions. Additionally, a future study in the domain of TL can go in a variety of areas such as:

- To begin, TL techniques can be investigated further and applied to a broader range of applications. New ways are required to overcome knowledge transfer challenges in more complicated circumstances. IFor example, in real-world settings, the client source-domain data may come from a different organization. In this scenario, the question of how to transmit knowledge from the source domain while maintaining user privacy is crucial
- Secondly, determining ways to quantify the transfer of information across domains while avoiding negative transfer is crucial. Although few studies have been done on negative transfers, more systematic research is still needed [62].
- Thirdly, the validity of TL requires further research [63].

**Fig. 6:**
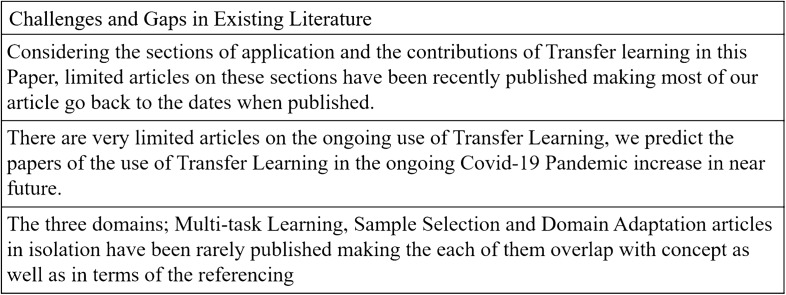
Challenges and gaps in the literatures of TL concerning this table

In terms of Challenges and gaps, the figure below depicts them (Fig. 6).

Furthermore, theoretical research can be performed to establish theoretical evidence for TL’s potency and validity. As a prominent and promising field of ML, TL has several advantages over classical/traditional ML, including reduced data need and less label reliance.

## Conclusion

TL is based on data distribution where one task is used in another. It uses outdated data and regulates the source task and target task. It follows some specific strategies based on data and model interpretation. This paper discussed the goals and strategies of TL by introducing the objectives and some of its learning approaches. In addition, we also briefly mentioned the techniques of TL at a model level, along with its applications. Several TL applications have been presented, such as in medicine, bioinformatics, transportation, recommendation, e-commerce, etc. The application of TL in numerous fields indicates that it is an essential research topic and can pave the way for the future technological era. However, it may seem difficult in practice.

## Data Availability

N/A (no data used).
